# HIV and HCV Activate the Inflammasome in Monocytes and Macrophages via Endosomal Toll-Like Receptors without Induction of Type 1 Interferon

**DOI:** 10.1371/journal.ppat.1004082

**Published:** 2014-05-01

**Authors:** Michael A. Chattergoon, Rachel Latanich, Jeffrey Quinn, Matthew E. Winter, Robert W. Buckheit, Joel N. Blankson, Drew Pardoll, Andrea L. Cox

**Affiliations:** 1 Department of Medicine, Johns Hopkins University School of Medicine, Baltimore, Maryland, United States of America; 2 Department of Oncology, Johns Hopkins University School of Medicine, Baltimore, Maryland, United States of America; Aarhus University, Denmark

## Abstract

Innate immune sensing of viral infection results in type I interferon (IFN) production and inflammasome activation. Type I IFNs, primarily IFN-α and IFN-β, are produced by all cell types upon virus infection and promote an antiviral state in surrounding cells by inducing the expression of IFN-stimulated genes. Type I IFN production is mediated by Toll-like receptor (TLR) 3 in HCV infected hepatocytes. Type I IFNs are also produced by plasmacytoid dendritic cells (pDC) after sensing of HIV and HCV through TLR7 in the absence of productive pDC infection. Inflammasomes are multi-protein cytosolic complexes that integrate several pathogen-triggered signaling cascades ultimately leading to caspase-1 activation and generation pro-inflammatory cytokines including interleukin (IL)-18 and IL-1β. Here, we demonstrate that HIV and HCV activate the inflammasome, but not Type I IFN production, in monocytes and macrophages in an infection-independent process that requires clathrin-mediated endocytosis and recognition of the virus by distinct endosomal TLRs. Knockdown of each endosomal TLR in primary monocytes by RNA interference reveals that inflammasome activation in these cells results from HIV sensing by TLR8 and HCV recognition by TLR7. Despite its critical role in type I IFN production by pDCs stimulated with HIV, TLR7 is not required for inflammasome activation by HIV. Similarly, HCV activation of the inflammasome in monocytes does not require TLR3 or its downstream signaling adaptor TICAM-1, while this pathway leads to type I IFN in infected hepatocytes. Monocytes and macrophages do not produce type I IFN upon TLR8 or TLR7 sensing of HIV or HCV, respectively. These findings reveal a novel infection-independent mechanism for chronic viral induction of key anti-viral programs and demonstrate distinct TLR utilization by different cell types for activation of the type I IFN vs. inflammasome pathways of inflammation.

## Author Summary

Pathogens are detected by the immune system in multiple ways that initiate responses to control infection. Two systems of first line defense against viruses are 1) the production of Type I interferons and 2) production of the cytokines IL-1β and IL-18 by the inflammasome. Type I interferons promote an antiviral state in the infected host. Inflammasome cytokines induce inflammation, modulate adaptive immune responses, and have direct antiviral effects. While both are produced in response to the chronic human viral infections HIV and HCV, we demonstrate here that inflammasome activation does not require cell infection and that the mechanisms for viral sensing as well as cell types in which sensing occurs are distinct between the two viruses and between the type I interferon vs. inflammasome systems. The relative amount of sensing via these different mechanisms may affect the balance between antiviral and inflammatory responses to chronic infection.

## Introduction

Human immunodeficiency virus (HIV) and hepatitis C virus (HCV) are RNA viruses capable of causing chronic infection, with an estimated 35 million [1] and 170 million [2] people infected worldwide, respectively. The innate immune response to these viruses generates an antiviral state that alters downstream adaptive immune responses to HIV and HCV. A key component of innate antiviral immunity is induction of the type I interferon (IFN) cytokine family. Type I IFNs induce hundreds of genes that promote an antiviral state in infected and normal cells [3]. Further, type I IFNs can be produced in almost all nucleated cell types upon infection, underscoring the importance of the cytokine family in antiviral immunity.

Endosomal Toll-like receptors (TLRs) detect intracellular pathogens then signal to the nucleus to induce transcription of type I IFNs and other genes encoding antiviral and pro-inflammatory mediators. Recognition of HIV by TLR7, and to a lesser extent by TLR9, in plasmacytoid dendritic cells (pDC) results in IFN-α production [4], [5]. This process requires endocytosis and trafficking of virions to early endosomes [6]. Plasmacytoid dendritic cells are also the primary producers of type I IFNs in HCV infection and have been shown to respond to HCV infection in neighboring hepatocytes or hepatoma cells via TLR7 [7].

Several important inflammatory pathways activated by viral infections involve activation of inflammasomes. Inflammasomes are multi-protein cytosolic complexes that integrate several pathogen-triggered signaling cascades, leading to caspase-1 activation and generation of the pro-inflammatory cytokines IL-18 and IL-1β. Both HIV and HCV infection are associated with higher serum levels of IL-18 [8], [9]. While the anti-viral functions of IL-1β are better studied, IL-18 also amplifies innate immune system antiviral responses [10]. IL-18 also plays a role in other inflammatory conditions, several of which have accelerated courses in persons with HIV or HCV infection, including atherosclerosis and diabetes [11], [12]. These observations suggest that there is a balance between direct anti-viral effects and inflammation associated with these cytokines with resultant tissue damage when the balance leans toward “non-productive” inflammation in the absence of viral control.

Whereas the capacity of HIV and HCV to induce type I IFN antiviral responses has been well characterized, considerably less is known about inflammasome activation by these viruses. We discovered that the cellular targets of the virus resulting in productive infection, inflammasome activation, and type I IFN production are distinct. Here, we demonstrate activation of the inflammasome in human monocytes and macrophages by HIV and HCV virions in a mechanism that does not require productive infection of the monocytes, but is instead dependent on clathrin-mediated endocytosis. TLR 8 is required to sense HIV and activate the inflammasome while TLR7 senses HCV. In monocyte lineage cells, the inflammasome pathway is activated preferentially over type I IFN production by HIV and HCV even in the presence of TLRs conventionally linked to IFN-α activation by these viruses. These findings define a novel mechanism for induction of an antiviral innate response in chronic human viral infections and demonstrate that distinct TLRs sense these RNA viruses to activate the inflammasome and shape the innate immune system response.

## Results

### HCV and HIV infected plasma activate monocyte inflammasomes

**Figure 1:**
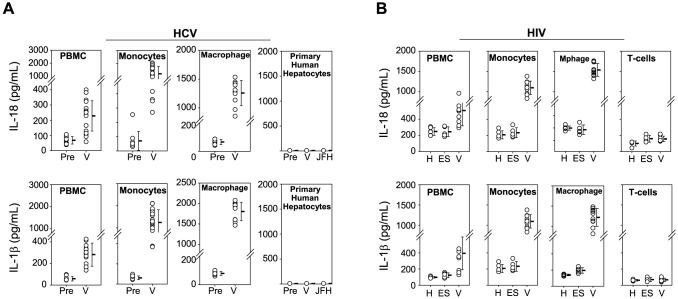
Monocytes produce inflammasome cytokines in response to HCV and HIV. (**A**) HIV and HCV uninfected PBMC, sorted monocytes, or *in vitro* differentiated macrophages were cultured with plasma from HCV infected subjects (V, *n = 15, plasma vol. creates at the final concentration of 5×10^5^ HCV RNA IU/mL in culture*) or plasma from the same subjects prior to infection (Pre, *n = 15, matched volume*). IL-18 and IL-1β were measured in duplicate after 24 h. Primary human hepatocytes were cultured with V and Pre plasma as well as the culture strain HCV_JFH-1_ (*n = 5*). Monocytes and derived macrophages secrete IL-18 (**top panel**) and IL-1β (**lower panel**) in response to V but not Pre plasma. Hepatocytes fail to produce inflammasome cytokines. In (**B**), IL-18 and IL-1β were measured after HIV and HCV uninfected PBMC, sorted monocytes, T-cells or *in vitro* differentiated macrophages were cultured with plasma from subjects on HAART (H, *n = 6*), plasma from elite suppressors (ES, *n = 5*), or viremic HIV plasma (V, *n = 15*). Plasma with measurable HIV stimulated significant monocyte IL-18 and IL-1β secretion. IL-18 produced from cells cultured with individual plasma samples (white circles), means (horizontal dash) ± S.D. are shown.

Previous research demonstrated that IL-18 and IL-1β are produced upon inflammasome activation in response to some acute viral infections [3], [10], [13], [14]. We previously reported marked serum IL-18 elevation during acute HCV infection, low-level IL-18 elevation during persistent infection, and normalization of IL-18 after spontaneous clearance of HCV [9]. Similarly, IL-18 is elevated in persons with uncontrolled HIV infection and successful HAART is associated with decreases in serum IL-18 [15], [16]. There are conflicting data in the literature about whether hepatocytes [17] or monocytes/macrophages [18], [19] are the source of IL-18 and IL-1β production in HCV infection. The source in HIV infection is in part CD4^+^ T cells, but CD4^+^ T cells require other cells or cytokines that remain unknown to produce IL-1β [20], [21]. To identify the cell types responsible for IL-18 and IL-1β production during HIV and HCV infection, we assessed the ability of multiple cell types to produce IL-18 and IL-1β in response to HIV and HCV. Pre-infection plasma (HCV seronegative, RNA negative) and plasma obtained from the same high-risk individuals during HCV infection/viremia (V) were cultured with normal PBMC, T-cells, B-cells, monocytes, myeloid dendritic cells, and plasmacytoid dendritic cells isolated from healthy HCV and HIV uninfected donors. HCV viremic plasma induced secretion of the inflammasome cytokines IL-18 and IL-1β from PBMC. Pre-infection plasma from the same individuals did not induce IL-18 or IL-1β (Figure 1A). Among PBMC constituents, we found monocytes to be the principle source of IL-18. T-cells, B-cells, and dendritic cells did not secrete any appreciable IL-18 when cultured with pre-infection or V plasma (Figure S1A). Monocyte derived macrophages also secreted IL-18 when cultured with V plasma (Figure 1A). In contrast, primary hepatocytes (the target cells of HCV infection) and hepatoma cell lines failed to produce IL-18 when cultured with this panel of HCV pre-infection and V plasma samples or with HCV_JFH-1_ despite productive HCV_JFH-1_ infection of Huh 7.5 and 7.5.1 cells (Figure 1A & Figure S1B). Thus, we find the major source of IL-18 to be monocytes and macrophages rather than the cellular targets of HCV infection, as has been shown *in vivo* and *in vitro* previously [19], [22].

We also cultured PBMC and PBMC subsets with plasma from HIV infected individuals with uncontrolled viremia (V), on HAART with undetectable HIV RNA, or elite suppressors (ES) exhibiting spontaneous immune control of HIV (Figure 1B & Figure S2). Monocytes and macrophages were again identified as the major source of IL-18 and IL-1β when cultured with viremic plasma and CD4+ T cells, the target of HIV infection, did not produce significant amount of either cytokine, consistent with previous data demonstrating that HIV infection of peripheral CD4^+^ T cells does not activate the inflammasome [21]. These data support monocytes/macrophages being a source of circulating IL-18 measured in the plasma of HCV and HIV infected individuals and a key cell type in which inflammasome activation occurs in HIV and HCV infection.

### Monocytes produce IL-18 when cultured with hepatitis C or HIV virions

**Figure 2:**
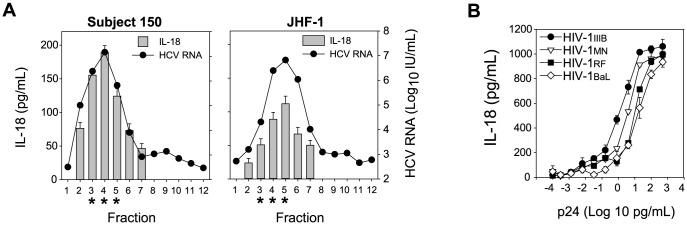
HCV and HIV virions stimulate monocytes to produce IL-18. HCV viremic plasma (**A**) or HCV_JFH-1_ (**B**) was separated into fractions by sucrose gradient equilibrium ultracentrifugation. HCV RNA (black circles) for each fraction was determined by RT-PCR. Monocytes are maximally stimulated to secrete IL-18 (grey bars, n = 3) when cultured with fractions in the density range 1.09–1.16 g/mL (*), which are most enriched in HCV RNA. (**c**) HIV_IIIB_, HIV_MN_, HIV_RF_ and HIV_BaL_ were cultured in activated CD4^+^ T-cells, p24 measured in supernatant, and supernatant transferred to monocytes. Monocytes cultured with HIV_IIIB_ (black circles), HIV_MN_ (white triangles), HIV_RF_ (black squares) and HIV_BaL_ (white diamonds) culture supernatant secrete IL-18 in a dose dependent manner. Symbols represent the mean ± S.D. of 6 experiments.

We next sought to determine whether virions or another plasma factor were responsible for inflammasome activation in monocytes. HCV virions were enriched from viremic plasma or HCV_JFH-1_ cell culture virus supernatant by sucrose gradient equilibrium ultracentrifugation [23]. We found that fractions in the density range 1.09–1.16 g/mL contained most HCV virions (as previously shown [23]) and were the most-stimulatory to monocytes (Figure 2A). In addition, filtration of viremic plasma through a 100 kD MCOW filter to remove virions but not soluble factors in excess of 100 kD, such as most cytokines, resulted in loss of inflammasome activation by the plasma (data not shown). To assess the role of HIV virions versus other plasma factors in inflammasome activation stimulated by plasma from HIV infected individuals, we added culture supernatant from four HIV strains to monocytes. Monocytes cultured with increasing amounts of virus produced IL-18 in direct correlation with the amount of HIV used as the stimulus and independent of HIV co-receptor usage pattern or strain tropism (Figure 2B). Virus purified from these preparations by ultracentrifugation through a 20% sucrose cushion demonstrate similar activity (data not shown). These data suggest that the HCV and HIV virions induce inflammasome activation rather than a cytokine or other factor in plasma.

### Inflammasome activation requires clathrin-mediated endocytosis of virus, not infection

**Figure 3:**
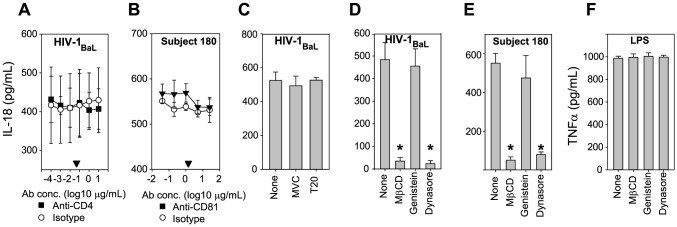
Inflammasome activation requires viral entry by clathrin-mediated-endocytosis. (**A**) The HIV entry receptor CD4 was blocked on monocytes with mAb prior to addition of HIV_BaL_ culture supernatant (HIV_BaL,)_. IL-18 production at multiple anti-CD4 mAb concentrations is shown relative to isotype control-treated monocytes. Symbols represent the mean ± S.D. of *n = 4* experiments, (black triangles represents the mAb concentration previously determined to prevent HIV infection of CD4+ T-cells. CD4 blockade did not alter IL-18 production from monocytes at concentrations >100 fold that required to block infection. (**B**) The HCV entry receptor CD81 was similarly neutralized using anti-CD81 mAb. CD81 blockade did not alter IL-18 production from monocytes cultured with plasma from HCV_Subject 180_ (HCV_Subject 180_). Symbols represent the mean ± S.D. of *n = 4* experiments. (**C**) Receptor-mediated HIV entry or fusion was inhibited by adding Maraviroc (MVC) or T20, respectively to cultures of HIV_BaL_ and monocytes. IL-18 production was measured after 24 h with no inhibition seen. Bars represent the mean ± S.D. of *n = 8* replicates. Clathrin-mediated or clathrin-independent endocytosis was inhibited by pre-incubating monocytes with MβCD, Genistein or Dynasore then adding (**D**) HIV_BaL_ or (**E**) HCV_Subject 180_. IL-18 production was significantly reduced by Dynasore and MβCD, suggesting the clathrin-mediated endocytosis of the virus is required for inflammasome activation by HCV and HIV. (**F**) LPS was added to treated cells and IL-18 or TNF-α production measured after 24 h to assess cellular function independent of inflammasome activation. Bars represent the mean ± S.D. of *n = 8* replicates, (*) denotes comparisons with p≤0.05 compared to the untreated cells.

We next investigated how HIV and HCV stimulate inflammasome activation. As one method of determining if inflammasome activation required infection, we blocked CD4, which is required for receptor-mediated HIV infection, prior to culturing monocytes with HIV virions. Anti-CD4 mAb had no effect on inflammasome activation even when added at concentrations >100 fold that required to block HIV infection of CD4 T cells (indicated by the arrowhead, Figure 3A). Similar results were obtained when three other HIV strains were blocked by anti-CD4 mAb or when HCV infected plasma was blocked by anti-CD81 mAb Figure 3B and Figure S3A and B). Figure 3B and Figure S3A and B). CD81 is the major entry receptor required for HCV infection of hepatocytes. These data suggest that the monocyte–virus interaction leading to inflammasome activation is not dependent on infection via the entry receptor of either virus. To confirm this, monocytes were cultured with HIV in the presence of the antiretroviral drugs Maraviroc (MVC, a CCR5 antagonist that blocks utilization of the HIV co-receptor CCR5), or T20 (an HIV fusion inhibitor). Regardless of the HIV strain used, neither antiretroviral drug when used at concentrations in excess of those required to prevent HIV infection of CD4^+^ T-cells altered IL-18 production, further supporting an infection independent mechanism of inflammasome activation by HIV (Figure 3C and Figure S3C).

We then sought to determine whether virion entry into the monocyte through endocytosis rather than infection is sufficient to activate the inflammasome. Treatment of monocytes with an inhibitor of caveolar-mediated endocytosis (Genistein) had no effect on inflammasome induction, while treatment with inhibitors of clathrin-dependent only (Methyl-β-cyclodextrin, MβCD) or of both caveolar and clathrin-mediated endocytosis (Dynasore) significantly decreased IL-18 production by HIV (Figure 3D, Figure S3D) and HCV (Figure 3E, Figure S3E). Monocyte TNF-α production in response to LPS was not altered with these treatments, suggesting a specific effect on inflammasome activation rather than cellular toxicity resulting in cells with more globally impaired function (Figure 3F). Thus, monocyte production of IL-18 was dependent on clathrin-mediated endocytosis, but not infection via entry receptors, consistent with an infection independent mechanism of inflammasome activation.

Inflammasome activation and secretion of mature IL-1β and IL-18 requires integration of two signals. The first occurs with virus sensing by a pattern recognition receptor (PRR) and results in increased transcription of inflammasome pro-cytokines including pro-IL-1β mRNA. The second is viral activation of a Nod-like receptor (NLR) resulting in downstream caspase-1 activation and cleavage of pro-IL-1β and pro-IL-18 into the mature proteins to be secreted. Given our data that endocytosis is required for activation, we focused on endosome located TLR3, 7, 8, and 9 as the most likely PRR to sense virus and generate signal 1.

### TLR8 is the primary inflammasome sensor of HIV while TLR7 senses HCV

**Figure 4:**
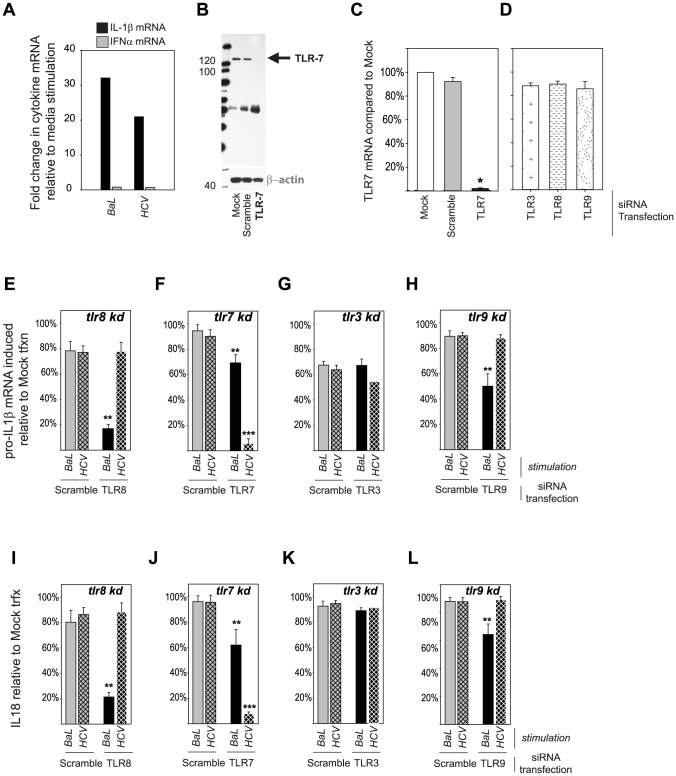
Differential importance of endosomal TLRs in inflammasome sensing of HIV and HCV. Monocytes were cultured without stimulation or with HIV_BaL_ or HCV_Subject 180_ and IL-1β, IFNα and measured at multiple timepoints. Fold change in expression of IL-1β (black bar) and IFNα (grey bar) following HIV_BaL_ or HCV_Subject 180_ relative to stimulation with media alone is shown at 6 h (**A**). Functional knockdowns of the endosomal located TLRs in monocytes were generated by RNA interference. Monocytes were mock transfected or transfected with either siRNA targeting TLR7 or a non-targeting sequence (Scramble). After 24 h, cell lysates were prepared and efficacy of knockdown determined by (**B**) western blot and (**C**) qRT-PCR. (**D**) Specificity of the TLR7 siRNA was confirmed by qRT-PCR using primers for TLR3, TLR7, TLR8, and TLR9. (*) denotes comparisons with p≤0.05 compared to the mock transfected cells. Monocytes in which endosomal TLR knockdown were generated were cultured with HIV_BaL_ (solid bars) or HCV_Subject 180_ (hatched bars) and pro-IL-1β mRNA transcription measured at 6 h (**E–H**) and IL-18 secretion measured at 24 h (**I–L**). Shown are the relative production of pro-IL-1β mRNA and IL-18 in TLR8 (**E**, **I**), TLR7 (**F**, **J**), TLR3 (**G**, **K**) and TLR9 (**H**, **L**) knockdown monocytes normalized to mock transfected monocytes (no siRNA) stimulated with the same viruses. Bars represent the mean ± S.D. for *n = 6–9* independent transfection experiments, (**) denotes comparisons with p≤0.05 and (***) denoted p≤0.001 compared to the scramble siRNA transfected cells.

In a previous study, pDCs were shown to produce type I interferon in response to TLR7 mediated sensing of HCV infected hepatoma cells. However, such stimulation of pDCs does not result in IL-18 or IL-1β production by pDCs ([7] and Francis Chisari, personal communication). We assessed the ability of monocytes to produce inflammasome cytokines and type 1 IFN after stimulation with both HIV and HCV and found that monocytes preferentially transcribe IL-1β over IFN-α. The relative abundance of the cytokine mRNA favors IL-1β by a factor >10^4^ (Figure 4A) with no IFN-α mRNA detected in resting cells. We next employed siRNA to knockdown all four endosomal TLRs in monocytes (verified by monocyte lysate Western blot and RT-PCR, Table S1, Figure 4B–D, Figure S4 and Figure S5) and repeated co-culture of the TLR-knockdown monocytes with HIV_BaL_ or purified HCV. Specificity of the method to selectively knockdown the TLR of interest was assessed by qRT-PCR, e.g., monocytes transfected with TLR7 siRNA only were also assessed for effects on TLR3, 8 and 9 (Figure 4D). Transcription of pro-IL-1β mRNA induced by HIV_BaL_ was dramatically decreased when TLR8 was knocked down (Figure 4E). Minimal effects were noted after knockdown of TLR7 or 3 and knockdown of TLR9 had a modest effect (Figure 4F–H). Similar effects were seen in production of mature IL-18 (Figure 4I–L). In contrast, with HCV stimulation, knockdown of TLR7 decreased pro-IL-1β mRNA levels by 95% (Figure 4F), while knockdown of TLR8 and 9 had no effect. Similar effects were seen in production of mature IL-18 (Figure 4J). Thus, the cell types that produce type I interferon and IL-18/IL-1β are distinct whether the same TLR or a distinct TLR senses virus.

**Figure 5:**
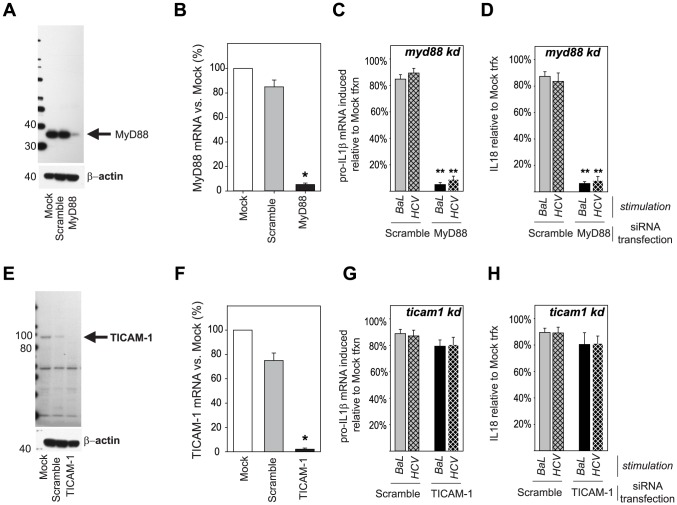
MyD88 is required for inflammasome sensing of HIV and HCV. Knockdowns of the TLR adaptors MyD88 (**A**, **B**) and TICAM-1 (TRIF, **E**, **F**) were generated and confirmed by previously described RNA interference techniques. (*) denotes comparisons with p≤0.05 compared to the mock transfected cells. Monocytes in which MyD88 was knocked down were cultured with HIV_BaL_ (solid bars) or HCV_Subject 180_ (hatched bars) and pro-IL-1β mRNA transcription measured at 6 h (**C**, **G**) and IL-18 secretion measured at 24 h (**D**, **H**). Shown are the relative production of pro-IL-1β mRNA and IL-18 in MyD88 (**C**, **D**) or TICAM-1 (**G**, **H**) knockdown monocytes normalized to mock transfected monocytes (no siRNA) stimulated with the same viruses. Bars represent the mean ± S.D. for *n = 6–9* independent transfection experiments, (**) denotes comparisons with p≤0.001 compared to the scramble siRNA transfected cells.

Except for TLR3, all TLRs depend at least in part upon the MyD88 adaptor protein for full signaling activity with TLR7 and 9 being completely MyD88 dependent [20]. In contrast, TLR3 does not signal through MyD88, but does so through the adaptor TICAM-1 [20]. Consistent with TLR8 (HIV) and 7 (HCV) being the relevant PRR for sensing and signal 1 generation, monocyte knockdown of MyD88 (Figure 5A–B) resulted in complete loss of pro-IL-1β mRNA (Figure 5C) and IL-18 (Figure 5D) production in response HIV and HCV. In contrast, monocyte knockdown of TICAM-1 (Figure 5E–F) (had no effect on generation of signal 1 or mature cytokines for either virus (Figure 5G–H), consistent with the lack of effect of TLR3 down. Having demonstrated that TLR8 and TLR7 are required for generation of signal 1 in response to HIV and HCV, respectively, we then sought to determine which NLR were necessary for generation of signal 2.

### NLRP3 assembly is required for inflammasome activation in response to HIV and HCV

**Figure 6:**
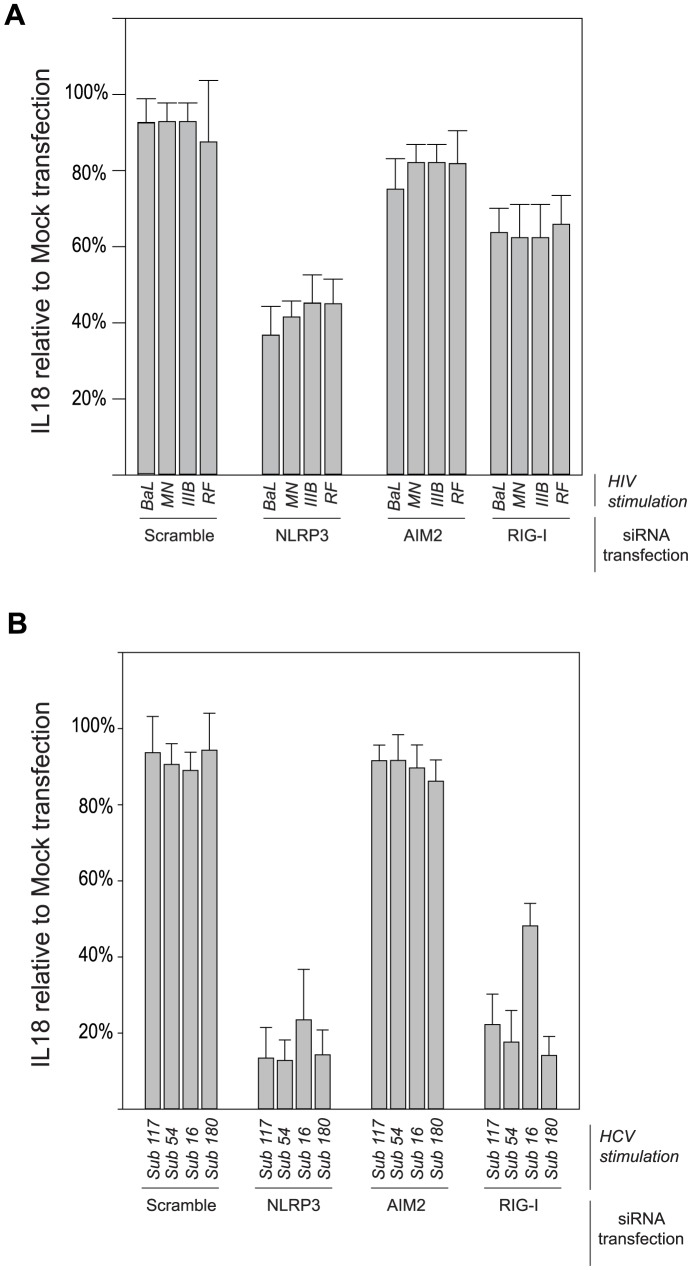
Differential importance of cytoplasmic sensors in inflammasome response to HIV and HCV. Functional knockdowns of the cytoplasmic sensors NLRP3, RIG-I and AIM2 in monocytes were created as previously described for TLRs. (**A**) Knockdown monocytes were cultured with HIV_BaL_, HIV_RF_, HIV_IIIB_, or HIV_RF_ and IL-18 measured after 24 h. IL-18 produced relative to that of mock transfected cells is shown. (**B**) Similarly prepared monocytes were cultured with a panel of plasma from HCV infected individuals (HCV_Subject 117_, HCV_Subject 54_, HCV_Subject 16_, HCV_Subject 180_) and IL-18 measured at 24 h. Bars represent the mean ± S.D. for *n = 6–9* independent transfection experiments.

NLRP3, RIG-I, and AIM2 are well-characterized intracellular inflammasome sensors. Caspase-1 activation and IL-18 and IL-1β maturation are triggered by the assembly of these inflammasome protein complexes in response to some acute viral infections [9]. In order to determine which of these inflammasome complex components assembled in monocytes in response to HCV or HIV, we used siRNA to knockdown NLRP3, RIG-I, or AIM2 in monocytes (Figure S6). We then cultured the modified monocytes with HIV culture strains (Figure 6A) or acute HCV infection plasma (Figure 6B, Figure S7). NLRP3 knockdown resulted in a 58±4% and 82±6% reduction in IL-18 production upon exposure to HIV or HCV, respectively, compared to the non-targeting sequence. For HCV, knockdown of RIG-I resulted in IL-18 reduction by a mean of 70±13%. RIG-I knockdown had less significant effect with HIV, reducing IL-18 production by 35±3% compared to the scramble sequence. Consistent with its known role as a DNA sensor, AIM2 knockdown had little effect on IL-18 production upon exposure to HCV (9±3%.), a positive sense single strand RNA virus with no DNA intermediate. In contrast, HIV has a DNA stage as a retrovirus but knockdown of AIM2 in monocytes minimally reduced IL-18 production compared to the non-targeting sequence 8±4%, consistent with sensing occurring prior to reverse transcription. NLRP3 had the largest and most consistent effect, indicating dependence on NLRP3 assembly for both viruses with RIG-I a less important sensor.

## Discussion

Innate sensing of viral infections results in both type I interferon production and in inflammasome activation. Chronic viral infections such as HCV and HIV induce a persistent inflammatory state and the mechanisms through which viral sensing induces activation have been unclear. Here, we demonstrate that both viruses preferentially activate the inflammasome but not type I IFN production in isolated monocytes and macrophages from PBMC and that the inflammasome is not activated in isolated cells infected by and in which the viruses replicate. Our data using HCV are consistent with two recent papers suggesting that inflammasome activation of liver resident macrophages in the liver result in inflammation and liver disease in HCV infection [18], [19]. However, one previous publication presented data suggesting that hepatocyte infection results in inflammasome activation, which our data do not support [17]. Previous publications used an HCV culture strain and a transformed macrophage cell line for the *in vitro* work rather than primary cells. Another publication required activation of the macrophage cell line with PMA to get inflammasome activation. The culture strain of HCV used is uniquely adapted for successful propagation in culture and may not be representative of natural HCV infecting strains. Our data were obtained using primary human cells and natural HCV strains isolated from infected subjects and are likely more representative of natural HCV behavior.

For HIV, Doitsh *et. al.* recently demonstrated in human lymphoid aggregate culture (HLAC) that when HIV enters quiescent CD4^+^ T cells but does not productively infect them (termed abortive infection), those CD4^+^ T cells undergo inflammasome activation and pyroptosis [20]. Pyroptosis is the process of cell death triggered by inflammasome activation. Monroe *et. al.* showed that the death of lymphoid CD4^+^ T cells abortively infected with HIV required the host DNA sensor interferon-gamma-inducible protein 16 (IFI16) [24]. A previous paper from the same group demonstrated that quiescent CD4 T cell death required other cells or cell factors in HLAC and that isolated peripheral CD4^+^ T cells did not undergo cell death upon abortive infection with HIV [21]. Our results showing that isolated CD4^+^ T cells do not undergo inflammasome activation in response to HIV are consistent with this. Whether HIV activates macrophages, as we demonstrate here, to provide the necessary additional factors for CD4^+^ T cell activation in HLAC or *in vivo* remains to be determined.

Our study also demonstrates that inflammasome activation by HIV and HCV requires clathrin-mediated endocytosis. While monocytes express CD81 and CD4, blockade of these primary receptors of HCV and HIV infection, respectively, is not associated with decreased inflammasome activation. Further, drugs that block HIV infection via virion fusion inhibition or CCR5 antagonism have no impact on inflammasome activation. This demonstrates that HIV and HCV activate the inflammasome in an infection independent mechanism that is dependent on clathrin-mediated endocytosis.

Using siRNA knockdown of endosomal TLR, we have shown that activation of the inflammasome depends on sensing of HIV by TLR8 and of HCV by TLR7 and that activation by both viruses depends on the adaptor protein MyD88 for generation of signal 1. Although the TLR required to sense HIV and generate the signal 1 needed to activate the inflammasome had not been defined previously, Lepelley *et. al.* demonstrated that HIV infected lymphocytes are potent inducers of type I interferon in pDC's via TLR7 [5]. Thus, both the cell type and TLR used differ for HIV-induced production of IFN-α and inflammasome activation with pDC sensing of HIV via TLR7 producing type I IFNs and monocyte/macrophage sensing of HIV via TLR8 producing IL-1β and IL-18. Consistent with our data in HCV, generation of signal 1 has been shown previously to be dependent on TLR7, although the importance of other TLR was not assessed in those papers [18], [19]. We also demonstrate that monocyte sensing of HCV and HIV does not result in production of type I interferon Rasaiyahh *et. al.* recently showed that HIV's use of cyclophilins and cleavage and polyadenylation specific factor 6 (CPSF6) in macrophages to cloak its replication may suppresses type I IFN production [25]. These new data combined with previous publications show that HCV and HIV activate the inflammasome in monocytes via TLR7 and TLR8, respectively, and type I IFNs in pDC via TLR7. Plasmacytoid DC do not produce IL-18 or IL-1β under conditions in which type I interferon is produced in response to HCV infection. Thus, cell type as well as the TLR used to sense virus determine whether type I interferon production or inflammasome activation will result. Further, the natural PAMPs recognized by TLR7 and TLR8 have not been well described [26]. In fact, there are few reports examining whether engagement of TLR7 and TLR8, which both recognize ssRNA, lead to different outcomes, and most focus on pDC [27]. While HIV has been shown to engage TLR8 in DC and activate NFκB, no association with inflammasome activation was demonstrated nor was this phenomenon studied in monocytes or macrophages [28]. We demonstrate functional differences in the innate immune response resulting from engagement of these two TLRs by natural PAMPs in monocytes. Heil *et. al.* showed that phosphothioate-protected RNA oligonucleotides from the U5 region of HIV-1 RNA induce DC and murine macrophages to produce TNF-α, interleukin-12p40, and IL-6 when complexed to cationic lipids known to facilitate the uptake of RNA [27]. However, their murine data show utilization of TLR7 rather than TLR8. In contrast, Heil *et. al.* show that the human cell line HEK 293 senses RNA oligonucleotides via TLR8 and note that ssRNA sensing may differ between species and cell types. We show that HIV is sensed by human macrophages and monocytes using TLR8 to result in inflammasome activation without IFN-α production. As in our data with human macrophages, Heil *et. al.* show that murine macrophages responded to oligonucleotides from HIV RNA without producing IFN-α.

For both HIV and HCV, we demonstrated through siRNA knockdown of key inflammasome sensors of pathogens that assembly of NLRP3 is required for generation of signal 2. NLRP3 is known to recognize RNA and has previously been shown to be the inflammasome component activated during monocyte infection with Influenza, Sendai, Adeno, Vaccinia, and Encephalomyocarditis viruses and upon macrophage contact with HCV [18], [19], [29]. RIG-I, another inflammasome sensor, had differential importance between the two pathogens, with RIG-I playing a more substantial role in HCV sensing. RIG-I is known to recognize 5′-triphosphate RNA and directly activate the inflammasome pathway. RIG-I also signals through the mitochondrial adaptor protein MAVS upon sensing HCV RNA and drives production of type I IFNs [30]. It thus has a role at the intersection of two distinct innate signaling pathways involved in HCV recognition, but it did not drive type I IFN production in monocytes in our experiments. Although it has been suggested that RIG-I can signal inflammasome activation directly, our demonstration of near complete loss of IL-18 production with NLRP3 blockade suggests that NLRP3 is required in RIG-I inflammasome signaling [29]. In sum, interaction of monocytes with HIV or HCV virions induces NLRP3 assembly and inflammatory cytokine production with variable use of other sensors.

Overall, we have demonstrated that activation of the inflammasome pathway in human monocytes and macrophages via HIV and HCV virions is infection-independent and dependent on clathrin-mediated endocytosis. This permits sensing of viruses regardless of their ability to infect monocytes and macrophages and may extend the capacity to sense viruses beyond the more limited subset that can productively replicate in those cells. TLR sensing differs between the two viruses but results in both viruses inducing inflammasome assembly in monocytes and IL-18/IL-1β production without type I interferon production. The cell specific factors that allow endosomal TLR sensing HIV and HCV to produce type I interferon in pDC and IL-1β and IL-18 in monocytes remain to be determined. However, the balance between antiviral and pro-inflammatory responses to infection may be determined in part by the differing response of these two cell types.

## Methods

### Ethics statement

All study protocols were approved by an IRB of the Johns Hopkins University School of Medicine. All adult subjects who provided blood samples also provided written informed consent, and a parent or guardian of any child participant provided written informed consent on their behalf.

### Collection of plasma from HCV or HIV infected individuals

Plasma samples collected longitudinally throughout HCV infections were obtained per protocol in the Baltimore Before and After Acute Study of Hepatitis [9] (BBAASH) cohort and characterized by subject number and as pre-infection (Pre) or viremic (V). HIV-infected plasma from persons with pharmacologic control of HIV (HAART), Elite suppressors demonstrating immunologic control (ES) of HIV or viremic (V) was collected from volunteers attending the Johns Hopkins Moore Clinic and characterized.

### Cells

The hepatoma cell lines Hep3B, HepG2 were purchased from ATCC (Manassas VA) and propagated per recommendations. Huh 7 and Huh 7.5.1 were obtained from Dr. Jake Liang (NIH) and Dr. Francis Chisari (Scripps Research Institute), respectively, and propagated as previously described [31]. Primary human hepatocytes (PHH) were obtained from Dr. Andrew Cameron (Johns Hopkins). PHH were plated on collagen-coated plates (Invitrogen, Grand Island NY) and cultured in DMEM supplemented with 15 mM HEPES pH 7.5 (Cellgro, Manassas VA), 2 mM L-Glutamine (Cellgro), ITS supplement (Sigma-Aldrich, St. Louis MO) and 10 mg/mL gentamicin.

Freshly collected, de-identified human blood Leuko Paks were obtained from the Johns Hopkins Blood Donor and Therapeutic Center. PBMCs were isolated by Ficoll-Hypaque gradient centrifugation. T-cells, B-cells, monocytes, plasmacytoid and myeloid dendritic cells were isolated by magnetic sorting per the manufacturer's protocol (Miltenyi Biotec, Auburn CA) and cultured in RPMI 1640 media (Invitrogen, Grand Island NY), 10% heat-inactivated human AB serum (Gemini, West Sacramento CA) and 2 mM L-Glutamine. The purity of sorted subsets was determined by flow cytometry using lineage specific antibodies.

### Antibodies

Antibodies anti-Human CD81 mouse mAb (clone 1.3.3.22, Santa Cruz Biotechnology, Santa Cruz CA); Anti-human CD4 mouse mAb (clone SK3) and an isotype control (clone MOPC-21, Biolegend, San Diego CA); Anti-human TLR3 rabbit mAb (clone D10F10, Cell Signaling Technology, Danvers MA); Anti-human TLR7 rabbit pAb (#2633S, Cell signaling Technology); Anti-human TLR8 rabbit mAb (clone D3Z6J, Cell Signaling Technology); Anti-human TLR9 rabbit mAb (clone D2C9, Cell Signaling Technology); Anti-human MyD88 rabbit mAb (clone D80F5, Cell Signaling Technology); Anti-human TRIF/TICAM-1 (clone MAB6216, R&D Systems, Minneapolis MN) mouse mAb; Anti-human NALP3 rabbit pAb (Imgenex, San Diego CA); Anti-human RIG-I (D14G6) rabbit mAb (clone D14G6, Cell Signaling Technology); Anti-human AIM2 rabbit pAb (Abcam, Cambridge, MA) and Anti-human Actin (A2066, Sigma-Aldrich) were purchased from respective vendors. Secondary Antibodies anti-mouse IgG-HRP goat polyclonal (HAF007, R&D Systems), anti-rabbit IgG HRP-linked (7074, Cell Signaling Technology) were also purchased.

### Expansion of HIV cell culture strains

The following HIV-1 strains were obtained through the AIDS Research and Reference Reagent Program, Division of AIDS, NIAID, NIH after initial contribution of HIV_RF_ [32] from Dr. Dean Winslow; HTLV-III_MN/H9_ [33] from Dr. Robert Gallo; and, HIV_BaL_ [34] from Drs. Suzanne Gartner, Mikulas Popovic and Robert Gallo. HIV_IIIB_ was obtained from Dr. Suzanne Gartner (Univ. Maryland). We cultured HIV isolates including HIV_MN_ and HIV_IIIB_ (T-cell tropic), HIV_BaL_ (primarily macrophage tropic) and HIV_RF_ (dual tropic) strains in activated CD4^+^ T-cells, measured p24 in the supernatants, then cultured the supernatants with monocytes. HIV strains were spinoculated (1200 *g*, RT, 2 h) at 2 ng/mL HIV p24 per 1×10^6^ CD4^+^ T-cells, previously stimulated with PHA for 2 days. Unbound virions were removed and cells cultured for 7 days prior to harvest of infectious culture supernatant. HIV p24 Ag was measured using the Alliance p24 ELISA kit (Perkins Elmer, Waltham MA) per manufacturer recommendations. Virions were pelleted through a 20% sucrose cushion at 106,000 *g*, 4°C, 2 h.

### Expansion of HCV cell culture strains

HCV_JFH-1_ was the obtained from Dr. Jake Liang (NIH) and expanded as previously described [35].

### Sucrose density-gradient concentration and purification of HCV

HCV_JFH-1_ culture supernatants and HCV positive plasma were centrifuged (400 *g*, 4°C, 5 min) to remove cellular debris then loaded onto a 20–60% sucrose gradient [36] (12 mL total volume) and centrifuged (135,000 *g*, 4°C, 16 h) in a AH-650 Swinging Bucket rotor (Thermo Scientific Sorvall, Asheville NC) in a Sorvall Discovery 100SE centrifuge. Twelve fractions (750 µL) were harvested sequentially from the top of the gradient, the density of each fraction was calculated and HCV RNA determined as previously described [9].

### Quantitative real-time PCR

RNA was isolated with the RNeasy Kit (Qiagen, Valencia CA) and used to synthesize cDNA with the Invitrogen Superscript III Reverse Transcriptase system. Quantitative PCR was run with primers (Table S2), premixed with FAM labeled probes (IDT) using Light Cycler 480 Probes Master mix (Roche). RNA input was normalized by concentration and HPRT was used as a housekeeping control. The ratio of target gene mRNA copies relative to HRPT mRNA copies was defined as Δ. Ratio of normalized mRNA copies among experimental conditions is defined as Δ/Δ and used to calculate the relative effect of the treatment.

### Western blot

Monocytes were lysed in Cell Lysis Buffer (Cell Signaling Technology) at a standard ratio of 5×10^7^ cells/mL following the manufacturer's protocol. Protein concentration was measured by BCA™ Protein Assay Kit (Thermo Scientific Rockford IL) to ensure consistency. For Westerns an equivalent of 2.5×10^5^ cells were loaded per well onto 4–12% or 7% SDS-PAGE gels and transferred to nitrocellulose using the iBlot system (Invitrogen). Nitrocellulose membranes were developed using the WesternBreeze Chemiluminescent Kit–Anti-Rabbit or –Anti-Mouse (Invitrogen) following the manufacturer's protocol.

### Cytokine measurements

Human IL-18, IL-1β, and TNF-α were measured with the Human IL-18 Platinum ELISA, Human IL-1β ELISA and Human TNF-α ELISA Ready-SET-Go kits respectively (eBiosciences, San Diego CA) using 50 µL of plasma or 100 µL of culture supernatant. Each sample was tested in duplicate. Data was acquired using a SpectaMax M5 (Molecular Devices, Sunnyvale CA). The LLOD of IL-18 was 40 pg/mL in plasma and 18 pg/mL in culture supernatant.

### siRNA gene knockdown in monocytes

siRNA targeting human TLR3 (s236, Life Technologies), TLR7 (SR308675, Origene), TLR8 (s27922, Life Technologies), TLR9 (SR310036, Origene), MyD88 (MYD88VHS404039, Invitrogen), TICAM-1 (s45113, Life Technologies), NLRP3 (SR314415, Origene), AIM2 (SR306264) and DDX58/RIG-I (SR309772, Origene) and a non-targeting scramble sequence were purchased. Monocytes were transfected by electroporation using the Human Monocyte Nucleofector Kit (VPA-1007, Lonza) following the manufacturer's protocol with the following modifications: for each transfection 2×10^6^ monocytes were transfected with 200 nM of the targeting siRNA. Transfected monocytes were cultured in ultra low-attachment 24-well plates (Corning). After 24 h, gene knockdown was confirmed by qRT-PCR and western blot.

### Identification of IL-18 producing cells

Hepatoma cell lines and PHH were plated at 6×10^4^ cells/well in 24-well plates (Becton Dickenson, Franklin Lakes NJ). PBMC or cell subsets were plated at 5×10^5^ cells/well in Ultra Low Attachment 96-well plates (Corning, Corning NY). Plasma from BBAASH subjects or HCV_JFH-1_ preparations was added to cultures to achieve a final HCV concentration of 5×10^5^ IU/mL. Matched volumes of the pre-viremic plasma were added to monocytes as a control. For viremic subjects, plasma IL-18 values were <500 pg/mL, but 0.3–2 microliters of plasma were used to stimulate the cells so the input IL-18 levels with dilution were <5 pg/mL. The plasma IL-18 levels were lower in seronegative/RNA negative individuals (<100 pg/mL) and matched volumes of plasma were used to stimulate PBMC's and sorted cells. At 24 and 96 h, culture supernatant was collected for cytokine measurements. Cells were lysed and total RNA harvested for RT-PCR experiments. HIV plasma was added to monocytes to create a RNA copy number of approx. 5–10×10^3^ copies/mL in the culture. Matched plasma volumes were used for the ES and HAART plasma. For cell culture strains each virus was titrated onto monocytes to determine optimal p24 concentrations for further experiments for the 4 HIV strains this ranged from 30–50 pg/mL p24.

### Modulation of IL-18 production by HIV entry inhibitors

HIV cell culture strains were pre-incubated with 10 µM of the CCR5 receptor antagonist Maraviroc (MVC) or 3 µM of the HIV fusion inhibitor Enfuvirtide (T20). T20 at 3 uM was found to inhibit 99.6% of HIV infection. MVC at 1.2 µM was found to inhibit 96.9% of HIV infection. The mixture was then added to monocytes and IL-18 production measured after 24 h.

### Modulation of IL-18 production by endocytosis inhibitors

Freshly isolated monocytes were incubated with inhibitors of clathrin-mediated endocytosis Methyl-β-cyclodextrin (5 µM MβCD, Sigma), caveolae/raft-mediated endocytosis Genistein (100 mM Sigma), or the dynamin inhibitor Dynasore (80 µM, Abcam, Cambridge, MA), concentrations previously determined to inhibit endocytosis of transferrin. Monocytes were stimulated with HIV or HCV at pre-determined concentrations or with LPS. IL-18 and TNF-α production measured after 24 h.

### Modulation of IL-18 production by knockdown of TLR pathway or inflammasome sensors

Gene knockdown was achieved by RNA interference as described above. Normal and modified monocytes were stimulated with HIV or HCV at pre-determined concentrations or with LPS. IL-18 and TNF-α production measured after 24 h. Parallel samples prepared in a similar manner were lysed at 6 h for qRT-PCR.

### Statistical analysis

One-way ANOVA and paired t-test were used to evaluate statistically significant differences between groups. Differences were considered statistically significant when p<0.05.

## Supporting Information

Figure S1 **HCV virions do not stimulate lymphocytes, dendritic cells, or hepatoma cell lines to produce inflammasome cytokines.** (**A**) HIV and HCV uninfected sorted T-cells, B-cells, myeloid dendritic cells (mDC) and plasmacytoid dendritic cells (pDC) were cultured with plasma from HCV infected subjects (V, *n = 15*) at the final concentration of 5×10^5^ HCV RNA IU/mL in culture or plasma from the same subjects prior to infection (Pre, *n = 15, matched volume*). IL-18 (**top panel**) and IL-1β (**lower panel**) were measured in duplicate after 24 h. As shown, the non-monocyte lymphocyte lineages fail to produce inflammasome cytokines. (**B**) Hepatoma cell lines (Huh 7.5.1, Huh 7, HepG2, Hep3B) were cultured with V (*n = 15*), Pre (*n = 15*) or culture strain HCV_JFH-1_ (*n = 5*) at the final concentration of 5×10^5^ HCV RNA IU/mL. Secreted IL-18 (**top panel**) and IL-1β was measured in duplicate at 24 h. Despite robust HCV production by HCV_JFH-1_ infected Huh 7.5.1 and 7 cells (data not shown), no hepatoma cell line produced IL-18 or IL-1β. IL-18 and IL-1β produced from cells cultured with individual plasma samples (white circles), means (horizontal dash) ± S.D. are shown.
(EPS)

Figure S2 **HIV plasma does not stimulate B-lymphocytes or dendritic cells to produce inflammasome cytokines.** (A) HIV and HCV uninfected sorted B-cells, myeloid dendritic cells (mDC) and plasmacytoid dendritic cells (pDC) were cultured with viremic HIV plasma (V, n = 15), plasma from subjects on HAART (HAART, n = 6) or elite suppressors (ES, n = 5). As observed previously, non-monocyte lymphocyte lineages fail to produce inflammasome cytokines IL-18 (top panel) and IL-1β (lower panel), which were measured in duplicate after 24 h. IL-18 and IL-1β produced from cells cultured with individual plasma samples (white circles), means (horizontal dash) ± S.D. are shown.
(EPS)

Figure S3 **HIV and HCV induced IL-18 production is dependent on Clathrin-mediated endocytosis and is not inhibited by entry receptor blockers.** (**A**) The HIV entry receptor CD4 was blocked on monocytes with mAb prior to addition of HIV viruses HIV_IIIB_, HIV_MN_, and HIV_RF_. IL-18 production at multiple anti-CD4 antibody concentrations is shown relative to isotype control treated monocytes. Symbols represent the mean ± S.D. of *n = 4* experiments, (black triangles) represents the mAb concentration previously determined to prevent HIV infection of CD4^+^ T-cells. CD4 blockade did not alter IL-18 production from monocytes at concentrations three or more logs above that required to block infection. (**B**) The HCV entry receptor CD81 was similarly neutralized using anti-CD81 mAb. CD81 blockade did not alter IL-18 production from monocytes cultured with plasma from HCV_Subjects 16, 117, or 54_. Symbols represent the mean ± S.D. of *n = 4* experiments. (*) represents the mAb concentration previously determined to prevent HCV infection of Huh 7.5.1 hepatoma cells. CD81 blockade did not alter IL-18 production from monocytes at any concentration. (**C**) CCR5 co-receptor-mediated HIV entry and fusion were inhibited by adding MVC or T20, respectively, to cultures of HIV_IIIB_, HIV_MN_, or HIV_RF_ and monocytes. IL-18 production was measured after 24 h. Bars represent the mean ± S.D. of *n = 8* replicates, (*) denotes comparisons with p≤0.05 compared to the untreated cells. (**D**) Clathrin-mediated or clathrin-independent endocytosis was inhibited by pre-incubating monocytes with MβCD, Genistein or Dynasore. HIV_IIIB_, HIV_MN_, or HIV_RF_ was added to treated cells and IL-18 production measured after 24 h. Bars represent the mean ± S.D. of *n = 8* replicates. (**E**) Clathrin-mediated or clathrin-independent endocytosis was inhibited by pre-incubating monocytes with MβCD, Genistein or Dynasore. HCV_Subject 16_, HCV_Subject 54_ or HCV_Subject 117_ plasma was added to treated cells and IL-18 production measured after 24 h. Bars represent the mean ± S.D. of *n = 8* replicates, (*) denotes comparisons with p≤0.05 compared to the untreated cells.
(EPS)

Figure S4 **Relative expression of endosome TLRs.** Monocytes were isolated and protein lysates prepared and immunoblotted with antibodies for TLR3, TLR7, TLR8 and TLR9. Samples were exposed to film for equivalent periods of time to demonstrate relative abundance of the protein in untreated cells.
(EPS)

Figure S5 **Knockdown of endosomal TLR.** Monocytes were mock transfected, transfected with siRNA targeting (**A, B**) TLR3, (**C, D**) TLR8 or (**E, F**) TLR9 or a non-targeting sequence. After 24 h, cell lysates were prepared and efficacy and specificity of knockdown determined by western blot (A, C, E) and qRT-PCR (B, D, F), (*) denotes comparisons with p≤0.05 compared to the untreated cells.
(EPS)

Figure S6 **Knockdown of inflammasome sensors.** Monocytes were transfected with siRNA targeting (**A**) NLRP3, (**B**) RIG-I (**C**) AIM2 or a non-targeting scramble sequence. After 48 h, cells were collected and relative mRNA abundance after knockdown measured by qRT-PCR. Target siRNA treatment resulted in a greater than 50 fold decline in mRNA levels versus scrambled siRNA for each sensor (left panels). Protein knockdown was confirmed by western blot analysis as shown (right panels), (*) denotes comparisons with p≤0.05 compared to the untreated cells.
(EPS)

Figure S7 **Knockdown of inflammasome sensors alters HCV activation of monocytes.** Functional knockdowns of the cytoplasmic sensors NLRP3, RIG-I and AIM2 in monocytes were created as previously described for TLRs. Knockdown monocytes were cultured with a panel of plasma from HCV infected individuals (HCV_Subject 175_, HCV_Subject 160_, HCV_Subject 19_, HCV_Subject 138_) and IL-18 measured at 24 h. Bars represent the mean ± S.D. for *n = 6–9* independent transfection experiments.
(EPS)

Table S1 **Relative abundance of TLR and TLR adaptor mRNA in primary monocytes.** Monocytes were sorted and immediately lysed for RNA extraction. qRT-PCR for each TLR and TLR adaptors was performed and compared to HRPT. ΔΔCT vs. TLR3 calculated to demonstrate the relative abundance of each TLR and adaptor in monocytes.
(DOCX)

Table S2 **Primers used in PCR.** Primer sets utilized for qRT-PCR analysis.
(DOCX)
