# Supplementary material for: HIV and HCV Activate the Inflammasome in Monocytes and Macrophages via Endosomal Toll-Like Receptors without Induction of Type 1 Interferon
Source: PLoS Pathog. 2014 May 1;10(5):e1004082. doi: 10.1371/journal.ppat.1004082 (PMC4006909; doi:10.1371/journal.ppat.1004082)
Supplement: Table S1 — Relative abundance of TLR and TLR adaptor mRNA in primary monocytes. Monocytes were sorted and immediately lysed for RNA extraction. qRT-PCR for each TLR and TLR adaptors was performed and compared to HRPT. ΔΔCT vs. TLR3 calculated to demonstrate the relative abundance of each TLR and adaptor in monocytes. (DOCX) [file ppat.1004082.s008.docx]

| - - - - 1. Target gene |  | - - - - 1. Relative abundance         2. *Gene* mRNA vs. TLR3 mRNA |
| --- | --- | --- |
| Toll-like Receptor 3 |  | 1 |
| Toll-like Receptor 7 |  | 66 |
| Toll-Like Receptor 8 |  | 110 |
| Toll-like Receptor 9 |  | 4 |
|  |  |  |
| Myeloid Differentiation Primary Response Gene (88) |  | 415 |
| Toll-like Receptor Adapter Molecule 1 aka TRIF |  | 58 |
