# Supplementary material for: HIV and HCV Activate the Inflammasome in Monocytes and Macrophages via Endosomal Toll-Like Receptors without Induction of Type 1 Interferon
Source: PLoS Pathog. 2014 May 1;10(5):e1004082. doi: 10.1371/journal.ppat.1004082 (PMC4006909; doi:10.1371/journal.ppat.1004082)
Supplement: Table S2 — Primers used in PCR. Primer sets utilized for qRT-PCR analysis. (DOCX) [file ppat.1004082.s009.docx]

| - - - - 1. Target gene | - - - - 1. Source | - - - - 1. Preformatted Assay ID or Primers |
| --- | --- | --- |
| Interleukin-1 beta | IDT | Hs.Pt.53a.24443432 |
| Interleukin-18 | IDT | HS.PT.53A.4466633 |
| IFNA1 | IDT | F 5’-CCTCGCCCTTTGCTTTACT-3’ |
|  |  | R 5’-GCATCAAGGTCCTCCTGTTATC-3’ |
| Hypoxanthine-guanine Phosphoribosyltransferase | IDT | Hs.Pt.39a.22214821 |
| Toll-like Receptor 3 | IDT | Hs.Pt.53a.15580897 |
| Toll-like Receptor 7 | IDT | Hs.Pt.53a.21371763 |
| Toll-Like Receptor 8 | IDT | Hs.Pt.53a.22925271 |
| Toll-like Receptor 9 | IDT | Hs.Pt.53a.15547417 |
| Myeloid Differentiation Primary Response Gene (88) | IDT | Hs.Pt.53a.28311527.gs |
| Toll-like Receptor Adapter Molecule 1 aka TRIF | IDT | Hs00706140_s1 |
